# Sex-specific effects of localized muscle fatigue on upper body kinematics during a repetitive pointing task

**DOI:** 10.1186/s12891-022-05566-5

**Published:** 2022-06-27

**Authors:** Chen Yang, Julie N. Côté

**Affiliations:** 1 Department of Kinesiology and Physical Education, McGill University, Montreal, QC H2W 1S4 Canada; 2 Occupational Biomechanics and Ergonomics Laboratory, Michael Feil and Ted Oberfeld/CRIR Research Centre, Jewish Rehabilitation Hospital, Laval, QC H7V 1R2 Canada

**Keywords:** Repetitive movement, Sex differences, Upper limb, Spine, Variability

## Abstract

**Background:**

Females are reported to have a higher risk of musculoskeletal disorders than males. Repetitive motions can lead to muscle fatigue, which may play a mediator role in the development of musculoskeletal disorders. However, sex differences in adaptations to localized fatigue at different joints are poorly understood. We examined the sex-specific effects of fatigue location on shoulder, elbow and spinal joint angles, and angular variabilities during a repetitive pointing task.

**Methods:**

Seven males and ten females performed a 30-s standing repetitive pointing task with their right upper limb when they were non-fatigued (NF), elbow-fatigued (EF), shoulder-fatigued (SF) and trunk-fatigued (TF), while trunk and upper body tridimensional kinematic data was recorded. Joint angles and angular variabilities of shoulder, elbow, upper thoracic spine, lower thoracic spine, and lumbar spine were calculated.

**Results:**

Results showed that shoulder angles changed the most after EF in males, but after SF in females. The similarities between sexes were that SF increased the variabilities at upper (lateral flexion: 0.15° greater than NF, rotation: 0.26° greater than all other conditions) and lower thoracic spine (lateral flexion: 0.13° greater than NF, rotation: averagely 0.1° greater than all other condition) in both sexes. TF altered upper thoracic spine variability (0.36° smaller than SF), lower thoracic spine angle (lateral flexion: 3.00° greater than NF, rotation: 1.68° greater than SF), and lumbar angle (averagely 1.8° smaller than all other conditions) in both sexes. However, females had greater lower thoracic spine angle (lateral flexion: 8.3° greater, *p* = 0.005) as well as greater upper (rotation: 0.53° greater, *p* = 0.006) and lower thoracic spine (rotation: 0.5° greater, *p* = 0.007; flexion: 0.6° greater, *p* = 0.014) angular variabilities than males.

**Conclusions:**

Results suggest that females’ fatigue responses focused on the trunk and spine. Results highlight a few sex differences in adapting to localized muscle fatigue, which may help explain how sex differences in repetitive motion-related injuries differ between joints.

## Background

Work-related musculoskeletal disorders (WMSDs) are part of the inflammatory and degenerative conditions that are caused or exacerbated by occupational work. According to the data published by Association of Workers’ Compensation Boards of Canada, in 2019, injuries to the trunk contributed to the most lost time claims (94,106), followed by injuries to the upper extremities (54,315) [1]. It is well known that WMSDs have created a huge economic burden and work time loss in Canada [2]. Statistics show that the lower back is affected the most by MSDs (affecting 577.0 million people in 2017) [3], followed by the shoulder. The elbow has lower but still considerable prevalence [4]. Moreover, women are shown to have higher injury prevalence, for all body parts. In a study on WMSDs prevalence among physical therapists, the prevalence of lower back WMSD was 22.9% in females and 9.4% in males. Similarly, 12% of females reported shoulder WMSD, while only 0.9% of males reported these complaints [4].

Repetitive motions are a known risk factor for WMSDs [5], and often lead to the development of muscle fatigue. Moreover, studies have provided experimental evidence for the role of muscle fatigue as a precursor to the development of WMSDs [6, 7]. Muscle fatigue can be characterized by an increased perceived effort and a decreased maximal voluntary muscle force, velocity of muscle contraction and relaxation, and power output, among other findings [8–10]. The effects of muscle fatigue can be quantified using quantitative approaches such as those that help quantify muscle electromyography (EMG) changes, body posture adjustments as well as inter-joint coordination and motor variability changes [10–13].

Using these approaches, Fuller et al. [12] found that body posture and shoulder kinematics were modified after fatigue in a standing pointing task. The movement-to-movement variabilities of shoulder and elbow motion amplitude were also found to increase after fatigue [14]. These studies also showed that muscle fatigue induced by the pointing task led to postural changes at other parts of the body such as to the trunk and elbow. Besides the postural changes, studies have also documented coordination adjustments with fatigue [13, 15, 16]. Previous studies have shown that with repetitive arm motion-induced fatigue, decreases in motion amplitude at fatigued joints were compensated by increases in motion amplitude and variability at other joints [13–16]. This has been interpreted as a manifestation of adjustments to the central motor command to modify the relative contributions of the degrees of freedom of the kinematic chain to prolong task performance. However, more study are needed to elucidate the mechanism underlying these kinematic adaptations to fatigue.

Other than fatigue, one’s sex is another risk factor that contributes to WMSDs [17, 18]. Studies have found that women have higher risks of upper body work-related pain and WMSDs [17, 18]. This is believed to be a result of differences in anthropometry, strength, flexibility, and other factors of biological origin [19–21]. In addition, previous studies showed that females use different biomechanical techniques compared to males during repetitive tasks [19–22]. For instance, females demonstrated higher upper body muscle activity than males in a painting task [23]. Also, Straker et al. [24] observed that females had a more upright habitual sitting posture while using a computer. In a manual dexterity task, women had higher upper trapezius and anterior deltoid muscle activation amplitudes and functional connectivity between neighboring upper limb muscles compared to men, regardless of the fatigue state [25]. Moreover, during occupational tasks, some authors have pointed out that females have different trunk and spinal kinematics [24, 26]. Plamondon et al. [26] discovered that the lumbar spine of females was close to full flexion when initiating a lifting movement, which might increase risk of back injuries. Another study revealed that females exhibited greater anterior pelvic tilt during computer work [24]. Accordingly, the anthropometry and flexibility differences in the spine of females and males may play a role in affecting spinal kinematics [27].

When it comes to muscle fatigue, in general, females are usually less fatigable than males for similar relative intensity of isometric fatiguing contractions [28–30]. However, this depends on the specific task [29]. The underlying physiological mechanism is thought to include sex differences in muscle mass, strength, blood flow perfusion, and fiber type proportion [31–34]. Interestingly, studies found that females and males adopt different movement patterns when muscle fatigue arises. Sex differences in fatigue adaptations might help explain the higher WMSDs risk in females. In a fatiguing upper limb task, the increase in trapezius muscle activation variability was found to be bigger in males than in females [35]. Besides, biceps activation variability decreased in males while it increased in females [35]. In a kinematic study, Bouffard et al. [36] showed that males decreased their humerothoracic elevation angle more while females increased humerothoracic elevation variability after fatigue in a standing pointing task. However, very few other studies focused on the sex-specific kinematic adaptation to muscle fatigue in dynamic tasks. With more studies, it may be possible to draw general conclusions that could help determine whether these sex differences in fatigue adaptations may help explain sex differences in mechanisms of WMSDs.

In real-life situations, fatigue may affect more than one joint at the same time, for instancewhen performing bouts of multi-joint tasks (e.g. lifting) during a work day. Most of the previous studies have only focused on fatigue around one segment of the body and only few studies have investigated the difference when different joints of the body were fatigued. Cowley and Gates [37] found that distal and proximal muscle fatigue brought different kinematic changes to the body during a standing wrenching task. Proximal fatigue elicited greater trunk leaning angle and elbow flexion angle, while distal fatigue caused earlier wrist extension and increased wrench velocity. Yang et al. [38] compared the kinematic adaptations to localized fatigue between shoulder, elbow and trunk during a standing repetitive pointing task. Results showed that shoulder fatigue brought the biggest overall kinematic change, and that trunk fatigue induced adjustments of trunk-shoulder coordination. In comparison, localized fatigue at the elbow led to changes at the shoulder and the trunk but no changes in coordination. However, to our knowledge, no previous study has compared how men and women may differ in these adaptations to localized muscle fatigue when performing a multi-joint task.

Therefore, the purpose of this study was to investigate the sex-specific kinematic adaptations to localized muscle fatigue during a standing repetitive pointing task when muscle fatigue was induced either at the shoulder, the elbow, or the trunk. We expected to see sex differences after shoulder fatigue that were similar to those of Bouffard et al. [36], where males had a smaller shoulder elevation angle and females had a greater shoulder elevation variability. We also expected to see sex differences after elbow fatigue, since females have less upper limb strength, but less sex differences after trunk fatigue, since there are less sex differences in trunk strength [39–41].

## Methods

### Participants

Seventeen right-handed healthy young adults (7 men, 10 women; age: men = 23.8 ± 2.3 (Mean ± SD) yrs, women = 22.3 ± 2.1 yrs, *p* = 0.23; height: men = 179.1 ± 5.3 cm, women = 166.9 cm ± 7 cm, *p* = 0.002; weight: men = 71.5 ± 5.2 kg, women = 55.9 ± 7.4 kg, *p* < 0.001) were recruited to participate in this study. The exclusion criteria were any previous experience in manual material handling work or any lower back pain, upper body injuries, musculoskeletal or cardiovascular impairment in the last 6 months before the data collection. All participants provided written informed consent prior to participation. The study was approved by the Research Ethics Board of the Centre for Interdisciplinary Research in Rehabilitation (CRIR) of Greater Montreal, and conducted in accordance with The Helsinki Declaration.

### Protocol

**Fig. 1:**
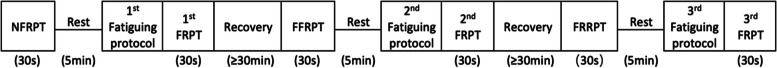
Schematic protocol of the study**.** NFRPT, FRPT, FRRPT stand for non-fatigued repetitive pointing task (RPT), Fatigued RPT, and Fatigue Recovered RPT, respectively. The 3 fatiguing protocols were those that fatigued the back, the shoulder, and the elbow, and were administered in random order for each participant

Before and after localized fatigue was to be induced at the low back, shoulder and elbow joints, participants performed a repetitive pointing task (RPT) as described in more details in Yang et al. [38]. Please refer to Fig. 1 for a flowchart of a sample entire experimental session. Briefly, the RPT requires that the participant, while comfortably standing, repetitively move their dominant (right) arm forward and backward, with their index finger alternatingly touching a target placed at 30%, and another one placed at 100% of arm reach, following the 2 Hz beat of a metronome [12]. Both targets were placed at each individual’s shoulder height, and their arm was also constrained to move entirely in a horizontal plane, at shoulder height, due to the presence of an obstacle placed under the elbow. The participant performed the RPT 4 times: when they were not fatigued, after the lower back muscles were fatigued, after the dominant shoulder was fatigued, and after the dominant elbow was fatigued [38]. After the instruction and practice of the RPT, the participant had 10 min of rest, after which they performed the RPT for 30 s as the non-fatigued RPT (NFRPT). The Rating of Perceived Exertion (RPE) of the shoulder, elbow, and lower back muscles were asked using the Borg CR-10 scale before and after the NFRPT [42]. Then, the participant sat on a chair and recovered for 30 min. Afterwards, series of isometric fatiguing tasks were performed to induce localized muscle fatigue one joint at a time, using isometric efforts performed until exhaustion (Borg CR10 scores of 10/10), as described in details in Yang et al. [38]. Briefly, fatigue was induced at the shoulder (deltoid and upper trapezius) by 1-min series, with 10 s breaks, of maintaining a horizontal (shoulder flexed 90 degrees and horizontally abducted 45 degrees) shoulder posture with the back strapped to a chair and a light weight strapped to the participant’s wrist (males: 1.4 kg; females: 0.7 kg), at the elbow (triceps branchii) by 1-min series, with 10 s breaks, of resisting an elbow flexor torque applied by a Theraband with the elbow flexed 35 degrees, and finally at the back (erector spinae) by 30 s series, with 10 s breaks, of maintaining a horizontal upper trunk at the edge of a table while their legs were secured to it. The order of the three fatiguing protocols was randomized using a customized randomization program in Matlab, and the total number of fatiguing trials at each joint was recorded for further analysis. Right after each fatiguing protocol, the participant performed another 30 s of the RPT as the Fatigued RPT (SFRPT for shoulder fatigued RPT, EFRPT for elbow fatigued RPT, TFRPT for trunk fatigued RPT). The RPEs of shoulder, triceps brachii and lower back muscles were asked using Borg CR-10 scale before and after each fatigued RPT. Between each fatiguing task, the participant sat on a chair and passively recovered for at least 30 min [43]. The Borg CR-10 exertion score of the target muscle was asked every 5 min during recovery until it went back to the same number as the one before the NFRPT. More details of this protocol can be found in Yang et al. [38].

### Data acquisition

A 7-camera motion capture system (MX3 VICON, Oxford Metrics Ltd., Oxford, UK) was used to record upper body kinematics (sampling frequency = 100 Hz) during each RPT trial. Passive reflective markers were placed on the forearm (lateral and medial epicondyle, most caudal-lateral point on the radial styloid, and most caudal-medial point on the ulnar styloid), humerus (acromioclavicular joint, lateral and medial epicondyle), C7, Incisura Jugularis, xiphoid process, upper thoracic spine (left and right transverse processes of T1, T6; placed 2.5 cm bilaterally from the spinous processes), lower thoracic spine (left and right transverse processes of T8, T12; placed 2.5 cm bilaterally from the spinous processes), lumbar spine (left and right transverse processes of L1, S1; placed 2.5 cm bilaterally from the spinous processes), and pelvis (left and right anterior superior iliac spine, greater trochanter and S1) [44].

### Data analysis

Kinematic data was low-pass filtered (digital 2^nd^ order Butterworth filter, cut-off frequency = 7 Hz, zero phase lag) in Visual 3D (C Motion, Germantown, MA). The segments of pelvis, trunk, right humerus, and right forearm were built in Visual 3D to calculate the angles of trunk, right shoulder, and right elbow [44, 45]. However, we modified the ISB recommendation for creating the humerus. We used the acromioclavicular joint instead of glenohumeral center of rotation to the create humerus joint coordinate system [38]. The rotation order of shoulder angles (humerus relative to thorax) was defined as followed: the first rotation is the plane of elevation; the second rotation is the elevation; the third rotation, internal/external rotation is the axial rotation angle. Elbow joint angle was calculated using Euler angles according to ISB recommendation [45]. Only elbow flexion/extension angle was used in further analysis. The segments of upper thoracic spine, lower thoracic spine, and lumbar spine were as described in Emery et al. [46]. The angles between upper and lower (UL) thoracic spine, lower thoracic spine and lumbar (LL) spine, lumbar spine and pelvis (LP) were calculated in Visual 3D. The rotation order was defined as follows: the first rotation was lateral flexion angle, second rotation was axial rotation angle, and third rotation was flexion/extension angle.

For each of those joint angles, data series collected during the RPT were partitioned into forward movement phases and backward movement phases using the wrist kinematic marker coordinates, and further analyses were done on only data of the forward movement phases. On average, there were 60 forward phases and 60 backward phases in each RPT trial. Data from each forward RPT movement phase was first time-normalized to 101 data points using Visual3D. The average value of the 101 points was calculated for each movement phase. Afterwards, the mean and SD of the averaged joint angle over all the forward movement phases (i.e., excluding data from the first and last 5 cycles to avoid accounting for incomplete cycles, and to avoid data boundary issues, i.e., cycles when the participant was accelerating to get into the rhythm, or decelerating to prepare to stop) were calculated to obtain the mean joint angles, and their movement-to-movement variability values [47]. Thus, each subject’s average angles represented the mean angle depicted during all of that participant’s forward movement phases of each 30 s RPT bout, and each participant’s angular variability outcome represented the movement-to-movement variability in that average joint angle.

### Statistical analysis

Two-way analyses of variance with repeated measurement were used to compare the total numbers of trials during elbow, shoulder, and trunk fatiguing tasks in females and males. For kinematic variables (joint angles, angular variabilities), generalized estimating equations (GEE) were used to examine the effects of Fatigue Location (NFRPT, SFRPT, EFRPT, TFRPT) and Sex (Male, Female). The GEE approach was selected because it has more power than repeated measures analyses of variance (RM-ANOVA), it is less restrictive in its assumptions than RM-ANOVA, it helps estimate the average change per group, and it is robust against a misidentified choice of correlation matrix [48, 49]. Fisher’s Least Significant Difference (LSD) tests were used to apply the pairwise comparisons of Sex and Fatigue Location. Benjamini–Hochberg procedures were applied to correct the p values and minimize type I error [50, 51]. The false discovery rate was set at 5%. Statistics were performed in Excel (Microsoft® Excel for Windows Version 15.26, Microsoft., US) and SPSS (SPSS Statistics v24, IBM Corp., US).

## Results

### Endurance time

On average, participants performed 9.18 ± 3.13 (Mean ± SD), 8.00 ± 1.90 and 8.88 ± 3.28 trials for elbow, shoulder and trunk fatigue, respectively. Specifically, for shoulder, elbow and trunk fatiguing protocol, females performed 7.80 ± 1.75, 9.00 ± 3.46 and 8.70 ± 3.92 trials, and males performed 8.29 ± 2.21, 9.43 ± 2.82 and 9.14 ± 2.34 trials. There was no difference of endurance time between the three fatiguing tasks (shoulder, elbow, and trunk; *p* = 0.16, *F* = 2.19) and between females and males (*p* = 0.79, *F* = 0.08) in any of those tasks.

### Shoulder angles

For the shoulder elevation angle, there was a significant interaction effect (*p* = 0.032) between fatigue location and sex. In males, the shoulder elevation angle was the smallest after EF than any other condition (NF: *p* < 0.0001; SF: *p* = 0.001; TF: *p* = 0.027). However, in females, the shoulder elevation angle was the smallest after SF compared to any other condition (NF: *p* < 0.0001; EF: *p* < 0.0001; TF: *p* < 0.0001). As for other shoulder angles, there were no sex*fatigue location interaction or sex main effects. However, there was a significant fatigue location effect on the plane of elevation angle. The plane of elevation angle after SF was the smallest compared to any other condition (NF: *p* = 0.011; EF: *p* < 0.0001; TF: *p* < 0.0001). In other words, the humerus was less forward-flexed after the SF. Finally, there were no significant effects on shoulder angle variability.

### Elbow angles

No sex*fatigue location interaction or sex main effects were detected on the elbow angles. There was a significant fatigue location effect on elbow flexion/extension angle. It was flexed the most after SF compared to any other conditions (2.61° more flexed than after NF, *p* = 0.001; 3.30° more flexed than after EF, *p* = 0.001; and 3.68° more flexed than after TF, *p* < 0.0001). As for its variability, there were significant main effects of sex and fatigue location on elbow flexion angular variability. Males had greater elbow flexion/extension variability than females (4.07° greater, *p* = 0.001). In addition, the elbow flexion angular variability was greater after TF compared to NF (1.32° greater, *p* = 0.002) and also compared to SF (1.38° greater, *p* < 0.01).

### Spinal angles

#### Upper thoracic spine (UL) angles

**Fig. 2:**
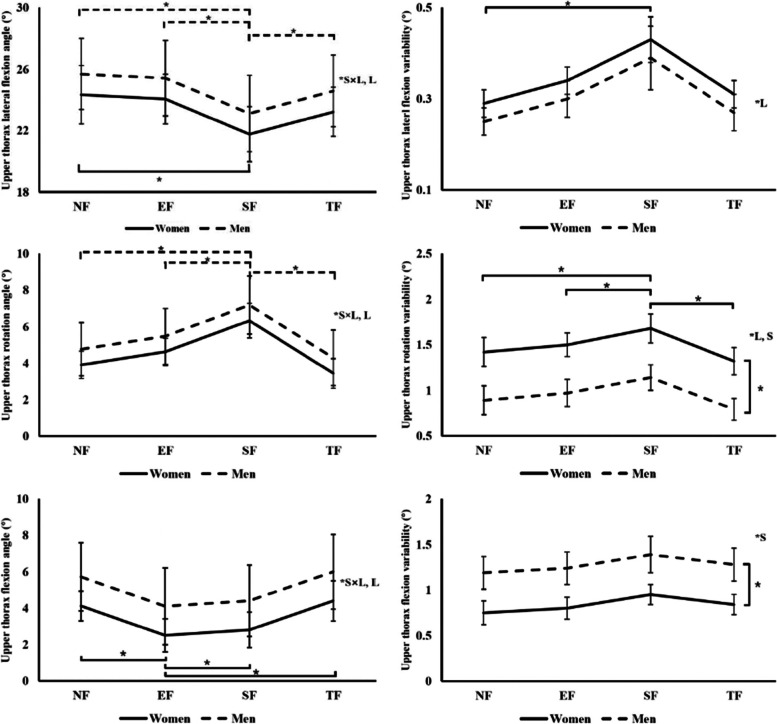
Upper thoracic spine angles and angular variabilities. The solid and dotted lines indicate joint angles and angular variabilities in women and men, respectively. The vertical bars above and below each point represent the standard deviation. The brackets and * indicate significant differences. “S”, “L”, “S × L” stand for significant effects of sex, fatigue location, and interaction between sex and fatigue location, respectively

There was a significant interaction (*p* < 0.0001) between sex and fatigue location on UL lateral flexion angle (Fig. 2). In males, the UL lateral flexion angle was the smallest after SF compared to all other conditions (4.01° smaller than NF, *p* < 0.0001; 3.75° smaller than EF, *p* < 0.0001; 3.21° smaller than TF, *p* < 0.0001). In females, however, the UL lateral flexion angle after SF was smaller than NF but not smaller than during the other conditions (1.29° smaller, *p* = 0.02). In other words, the upper thoracic spine was leaning towards the non-reaching side the least after SF in males, but for females, the leaning angle after SF was less than in NF. The interaction effect between sex and fatigue location (*p* < 0.0001) also existed on UL rotation angle. In males, the UL rotation angle was the greatest (upper thoracic spine was rotated the most towards the non-reaching side) after SF compared to any other condition (4.17° greater than NF, *p* < 0.0001; 3.58° greater than EF, *p* < 0.0001; 5.06° greater than TF, *p* < 0.0001). In females, the UL rotation angle was not significantly affected by fatigue location. In other words, males rotated the upper thoracic spine right more after SF while females showed no change. There was also an interaction effect between sex and fatigue location (*p* = 0.009) on UL flexion angle. In males, the UL flexion angle was the same in all conditions. However, in females, the UL flexion angle was the smallest (the upper thoracic spine was leaning forward the most) after EF compared to any other condition (1.77° smaller than NF, *p* = 0.002; 1.32° smaller than SF, *p* = 0.015; 2.65° smaller than TF, *p* = 0.001). As for the variabilities, the UL lateral flexion variability was greater after SF than NF (0.15° greater, *p* = 0.04). The UL rotation variability after SF was greater compared to all other conditions (0.26° greater than NF, *p* = 0.013; 0.17° greater than EF, *p* = 0.024; 0.36° greater than TF, *p* < 0.0001). Besides, females had greater UL rotation variability (0.53° greater, *p* = 0.006) and smaller UL flexion variability (0.44° smaller, *p* = 0.038) than males.

#### Lower thoracic spine (LL) angles

**Fig. 3:**
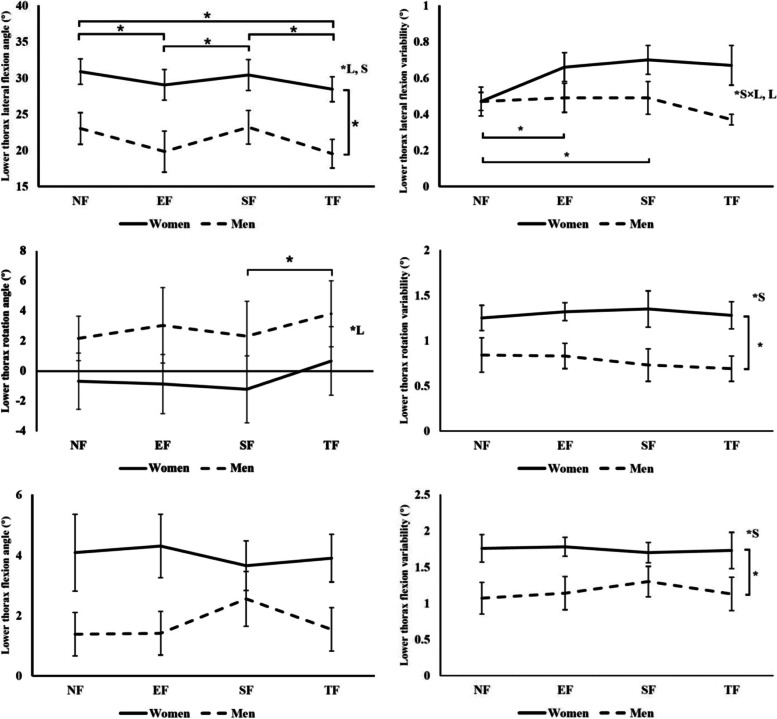
Lower thoracic spine angles and angular variabilities. The solid and dotted lines indicate joint angles and angular variabilities in women and men, respectively. The vertical bars above and below each point represent the standard deviation. The brackets and * indicate significant differences. “S”, “L”, “S × L” stand for significant effects of sex, fatigue location, and interaction between sex and fatigue location, respectively

There was no significant interaction effect between sex and fatigue on LL angles. However, there was a significant sex main effect (Fig. 3). Females had significantly greater mean LL lateral flexion angle than males (8.3° greater, *p* = 0.005). Moreover, there was a significant fatigue location effect (*p* < 0.0001) on the LL lateral flexion angle. The LL lateral flexion angle after SF was greater than after EF (2.3°, *p* = 0.0002) and TF (2.8°, *p* < 0.0001). It was also greater in NF than after EF (2.5°, *p* = 0.006) and TF (3.0°, *p* < 0.0001). This indicated that after EF and TF, the lower thoracic spine was leaning more towards the non-reaching side compared to NF. The LL rotation angle was greater after TF than after SF (1.7°, *p* = 0.007). As for the variabilities, there was an interaction effect between sex and fatigue location on LL lateral flexion variability. In males, there was no significant fatigue location effect. In females however, the LL lateral flexion variability in NF was smaller than it was after SF (0.2° smaller, *p* = 0.0005) and after EF (0.2° smaller, *p* = 0.0004). Besides, the LL rotation (0.5° greater in females, *p* = 0.007) and flexion (0.6° greater in females, *p* = 0.014) variabilities were greater in females than they were in males.

#### Lumbar (LP) angles

**Fig. 4:**
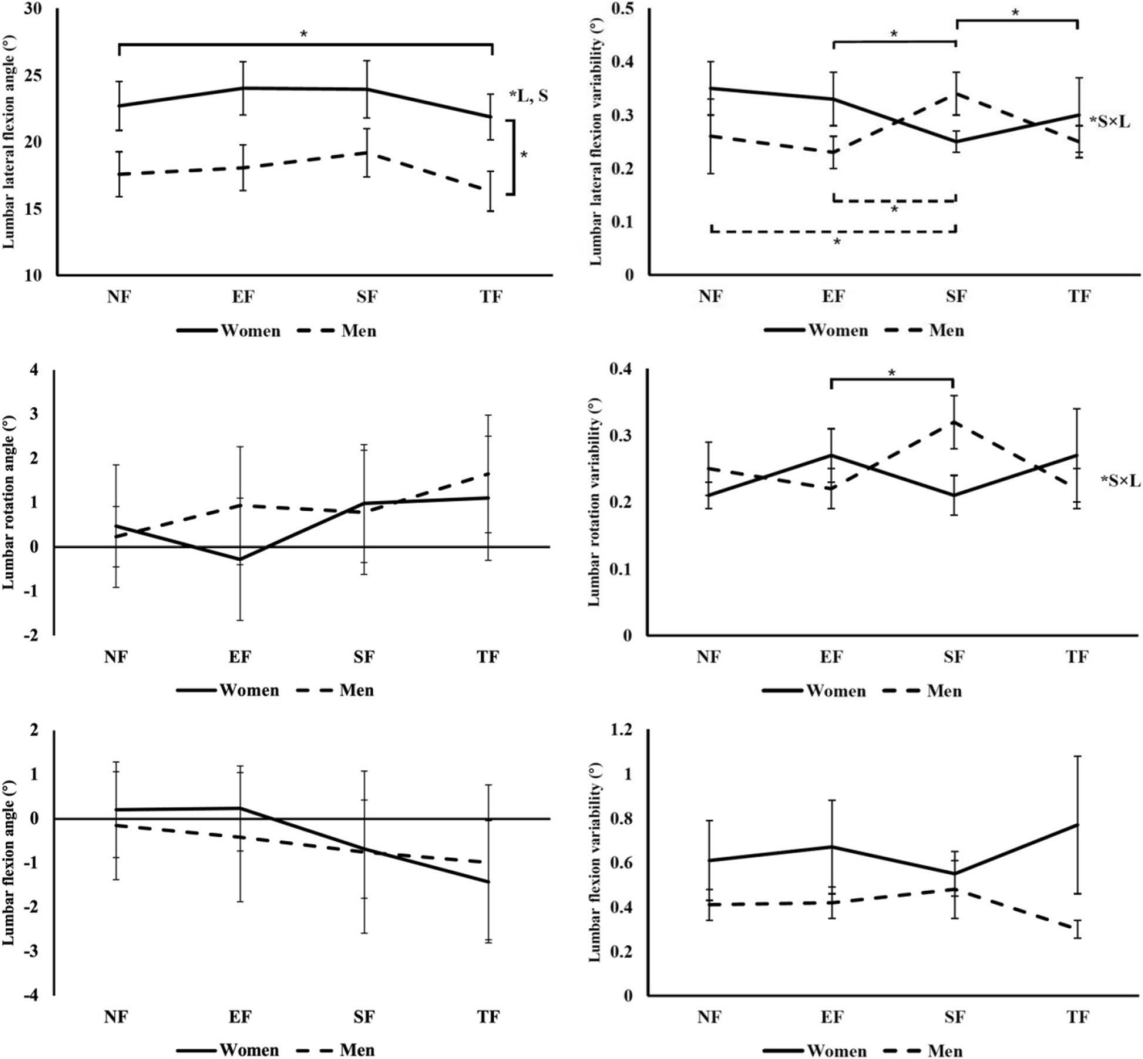
Lumbar angles and angular variabilities. The solid and dotted lines indicate joint angles and angular variabilities in women and men, respectively. The vertical bars above and below each point represent the standard deviation. The brackets and * indicate significant differences. “S”, “L”, “S × L” stand for significant effects of sex, fatigue location, and interaction between sex and fatigue location, respectively

No significant interaction between sex and fatigue location on LP angles was detected (Fig. 4). As for the fatigue location effect, the only significant joint angle change was the LP lateral flexion angle, which was smaller after TF than all other conditions (1.1° smaller than NF, *p* = 0.02; 2.0° smaller than EF, *p* < 0.0001; 2.5° smaller than SF, *p* < 0.0001). This implied that the lumbar segment was leaning towards the non-reaching side the least after the TF. In addition, there was a significant sex difference on the LP lateral flexion angle. Males had greater LP lateral flexion angle than females (5.4° greater, *p* = 0.032). This indicated that males leaned their lumbar region more towards the non-reaching side than the females. As for angular variabilities, there were interaction effects between sex and fatigue location on LP lateral flexion angle variability (*p* = 0.001) and LP rotation angle variability (*p* < 0.0001). In males, the LP lateral flexion variability was greater after SF than after EF (*p* = 0.02) and TF (*p* = 0.02), whereas in females, it was smaller after SF than after EF (*p* = 0.025) and TF (*p* = 0.025). Besides, the LP rotation variability in males was greater after SF than after EF (*p* = 0.003). But in females, it remained the same in all fatigue location conditions.

## Discussion

This study assessed whether there are sex differences in the effects of localized muscle fatigue on upper body kinematics during a repetitive upper limb task. During the fatiguing protocols, the resistance was controlled so that the male and female participants reached the same perceived fatigue level in a similar amount of time. Our results show that despite some similarities between sexes, females and males showed some differences in how localized fatigue affected how they accomplished the repetitive pointing task, especially in the spinal joint angles. More specifically, in agreement with our hypotheses, we showed sex differences after elbow fatigue, with females rotating their lower thoracic spine towards the non-reaching side more than males did. Besides, males changed their trunk and shoulder angles the most after elbow fatigue compared to the other fatigue conditions investigated in this study. However, contrary to our hypotheses, we showed that males had greater shoulder elevation than females after shoulder fatigue.

### Interaction effects of sex and fatigue location

Sex and fatigue location had interaction effects on shoulder and upper thoracic spine angles as well as spinal angular variabilities. For shoulder angles, the present study revealed that males were mostly affected by EF, while females were mostly affected by SF. Our results showed that after EF, males dropped their humerus the most, while females had similar shoulder adaptations after SF. Hunter revealed that males had greater EMG amplitude increases in their elbow flexors in an isometric elbow fatiguing task [28]. This could help explain our findings in that males’ elbow muscles might be more fatigued after the elbow fatiguing protocol, leading to their greater kinematic adaptations compared to SF and TF. Second, studies have shown that females and males react to shoulder muscle fatigue differently, using different fatigue adaptations. The shoulder, in particular, is a complex joint with many degrees of freedom, and previous studies have shown sex differences in the activation of agonists vs synergists during a fatiguing push-up task [52]. Moreover, Srinivasan et al. [35] observed that males had greater trapezius EMG variability than females during the performance of a fatigued repetitive pointing task similar to the one used in the present study. Bouffard et al. [36] also provided evidence for greater fatigue-related changes in males, where authors detected greater humerothoracic angle decreases in males than in females executing the same task. Thus, males and females may modify different shoulder parameters when adapting to fatigue. which could in turn have an impact on the shoulder angles as measured in the current study. Especially for spinal angles, males changed the upper thoracic spine angle the most at SF compared to all other fatigue locations, whereas females reacted the most to SF. Finally, only females increased lower thoracic spine variability after SF and EF, and only males increased lumbar variability after SF. Together, these results further support that females and males adapt to localized fatigue (e.g. SF) by utilizing different motor pattern and altering different body parts.

### Sex difference regardless of the fatigue location

Some kinematic differences between males and females were detected regardless of the fatigue location. For instance, the lower thoracic spine and lumbar lateral flexion angle were greater in females than males (meaning that females were leaning more on the moving arm’s side). As for upper thoracic spine lateral flexion angle, it was generally greater in males than in females. This might be a result of anthropometrical differences or due to the kinematic strategy that females and males adopted. In a study by Peharec et al. [20], the authors detected that pelvis range of motion is affected by sex, illustrated by female subjects having greater vertebral arcs than males in lateral flexion. Our results suggest that females tended to recruit the degrees of freedom at the lower thoracic spine while males tended to recruit more of the upper thoracic spine. In the study by Srinivasan et al. [35], the authors found that males showed greater trapezius activation variability than females did after fatigue, which also supports our finding of males altering their upper spinal kinematics. We observed that males had greater elbow movement variabilities; however, females had greater variabilities at upper the thoracic spine, lower thoracic spine, and lumbar regions. One interpretation would be that that females possessed more unstable spinal movement than males, while males showed more unstable arm movement than females during the RPT, although this could also reflect sex difference in other features like stiffness and flexibility. In another study also using an RPT task, Bouffard et al. [36] observed a greater elbow flexion variability in females than males, which is contrary to our results. This might be explained by slight differences between the RPT tasks in these two studies. In the current study, the movement frequency was doubled and the participant was holding a weight in their moving hand while performing the RPT. However, more studies are needed to further explain the spinal kinematic differences between the sexes when performing upper limb tasks.

### Fatigue location effect on spinal kinematics adaptation regardless of sex

In the present study, we further separated the spine into upper thoracic spine, lower thoracic spine, and lumbar segment, and calculated the relative angles between spinal sections. The results showed that TF had the greatest overall impact on the spinal kinematic adaptations. It altered the lower thoracic spine and lumbar angles as well as upper thoracic spine angular variability. Previous studies using a similar repetitive pointing task showed the important role of the trunk in adapting to fatigue induced by repetitive motions [11, 13, 36]. As for fatigue induced locally, Yang et al. [38] did not observe trunk angle changes during the post-fatigue RPT after TF, but this could be due to not having partitioned the trunk in different sections in our previous study, since in the present study, we showed that TF altered the lower thoracic spine and lumbar angles. To our knowledge, this is the first study to show how localized trunk fatigue affects different spinal angles during a repetitive upper limb movement. Yang et al. [38] revealed that EF led to greater trunk lateral flexion angle. In our study, we further explained the EF effects on trunk angle where EF resulted in greater lower thoracic spine lateral flexion angle. Our results suggest that distal fatigue can affect proximal joint kinematics in a multi-joint movement. Moreover, it also implied that the spine can be adjusted at different sections to compensate for different localised fatigue. As for SF, previous studies using the RPT have shown the effects of SF on joint angular variabilities [12, 13, 35]. Bouffard et al. [36] detected increased trunk variabilities in three planes at the end of the RPT. Yang et al. [38] revealed that the trunk variabilities increased in two planes after SF. The present study showed that it was the variabilities of upper thoracic spine and lower thoracic spine, but not at the lumbar region, that increased. Since the lumbar segment is closer to the center of mass (CoM), this may suggest that SF impaired the upper thoracic spine but not the lower thoracic spine so that the CoM could be maintained. In the study by Fuller et al. [13], researchers found results to suggest that CoM variability was preserved so that the task performance could be performed successfully, a finding that our current results seem to support.

### Limitations

Even though some different kinematic changes were detected between females and males, the small sample size of this study cannot be ignored. The data in the current study was collected for a different project [38]. Before conducting the new data analysis, we ran a power analysis using G*Power (Version 3.1). When the Repeated measured ANOVA (RM-ANOVA) model was set, and effect size and power values were set at 0.25 and 0.8, the total sample size needed was 24. However, we only had access to data of 17 participants. To solve the issue of small sample size, we decided to use generalized estimating equations (GEE) to replace RM-ANOVA. The current study has an exploratory character and provides information for future studies with greater sample size to examine sex differences in fatigue adaptations. Secondly, we asked the participants to stand comfortably during the RPT. Nonetheless, there is a possibility that upper back angles may vary between participants, which we did not control for. Besides, since the goal of this study was to analyze whole-body kinematics including, for the first time in studies using the RPT, measurement of different sections of the back, we did not track scapula kinematics, even though shoulder fatigue might lead to changes in the thoraco-humeral joint center position, and further increase the potential error of shoulder kinematic measurements. Thirdly, an inherent sex difference in how shoulder fatigue was induced may represent another limitation. One piece of weight (0.7 kg) was inserted to the female’s wrist band while the male participant had two pieces (1.4 kg) during the shoulder fatiguing protocol. The weight and fatiguing task intensity were not normalized according to the participants’ muscle strength but were based on pilot studies, with a goal of obtaining comparable endurance times in the different localized fatigue protocols, which we were able to achieve. Nevertheless, the short durations of our experimental and fatiguing tasks limit the extent to which our results can be extrapolated to workplace-based fatigue and injury mechanisms.

### Perspectives and significance

The findings of this study showed similarities and differences between the sexes in how they adapt to fatigue. These results suggest that females and males might be placed at similar injury risk for some body parts but different risk level for other body parts. These results may have important consequences on jobs whose workforce may contain both males and females. For instance, since elbow fatigue had the greatest impact on males, but shoulder fatigue had the greatest impact on females, it may be necessary to allow more time between elbow efforts and multi-joint tasks for males, and more time between shoulder efforts and multi-joint tasks for females. Another strategy would be to adopt different work sequences in jobs that combine different tasks that lead to localized muscle fatigue for male and female employees. Future research may include larger amount of data collected in real workplaces to provide more ecological information of sex differences in fatigue adaptations.

## Conclusion

This study showed that females and males adapted to elbow and shoulder muscle fatigue differently. Males leaned and rotated the upper thoracic spine to the non-reaching side when the shoulder muscle was fatigued. Females adopted the same kinematic compensation pattern when the elbow muscle was fatigued. Females had greater upper thoracic spine and lower thoracic spine variability in multiple planes. Conversely, males had greater elbow flexion variability. Finally, spinal kinematics were altered differently to adapt to muscle fatigue at different body locations, regardless of sex. Future studies are needed to better understand the origin of the sex differences in kinematic adaptations to different muscle fatigue locations, and to estimate whether these differences may help explain the known sex differences in workplace injuries.

## Abbreviations

CoM: Center of mass
CRIR: Centre for interdisciplinary research in rehabilitation of greater montreal
EF: Elbow-fatigued
EMG: Electromyography
FRQS: Fond de recherche du québec – santé
GEE: Generalized estimating equations
ISB: International society of biomechanics
LL: Lower thoracic spine—lumbar
LP: Lumbar—pelvis
NF: Non-fatigued
RM-ANOVA: Repeated measured analysis of variance
RPE: Rating of perceived exertion
RPT: Repetitive pointing task
SD: Standard deviation
SF: Shoulder-fatigued
TF: Trunk-fatigued
UL: Upper thoracic spine – lower thoracic spine
WMSDs: Work-related musculoskeletal disorders

## Declarations

### Availability of data and materials

The datasets used and/or analyzed during the current study are available from the corresponding author on reasonable request.
